# Microwave assisted synthesis of some new thiazolopyrimidine and pyrimidothiazolopyrimidopyrimidine derivatives with potential antimicrobial activity

**DOI:** 10.1186/s13065-018-0419-0

**Published:** 2018-05-05

**Authors:** Ayman M. S. Youssef, Ahmed M. Fouda, Rasha M. Faty

**Affiliations:** 1 Department of Chemistry, College of Science, King Khalid University, Abha, Saudi Arabia; 2 Chemistry Department, Faculty of Science, Fayoum University, Fayoum, Egypt; 3 Department of Chemistry, Faculty of Science, Cairo University, Giza, 12613 Egypt

**Keywords:** Microwave-assisted technique, Biginelli reactions, Thioxopyrimidines, Thiazolo[3,2-*a*]pyrimidines, Thiazolopyrimidopyrimidine, Antimicrobial activity, Fluorescence

## Abstract

**Background and objective:**

A series of thiazolopyrimidine derivatives have been synthesized via multicomponent reaction and tested for biological activities. This research aims to develop a new synthetic method of poly fused pyrimidines under microwave irradiation. 6-Amino-4-aryl-2-thioxo-1,2,3,4-tetrahydropyrimidine-5-carbonitriles reacted with bromomalono-nitrile to give 3,7-diamino-5-aryl-5*H*-thiazolo[3,2-a]pyrimidine-2,6-dicarbonitrile more willingly than the isomeric 7*H*-thiazolo[3,2-*a*]pyrimidines. Thiazolopyrimidine derivatives reacted with carbon disulphide to produce 11-aryl-11*H*-1,2,3,4,7,8,9,10-octahydropyrimido[4″,5″:4′,5′]thiazolo[3′,2′-*a*]pyrimido[4,5-*d*]pyrimidine-2,4,8,10-tetrathione. The above mentioned reactions were established by using both conventional methods and microwave-assisted irradiation.

**Conclusion:**

This work provides a new method for preparing poly fused pyrimidines. The microwave-assisted technique is preferable due to the yield enhancements attained, time saving, and environmental safety reactions. The newly prepared compounds were verified for their antimicrobial activities. Also, the absorption and emission of some of the prepared compounds were studied.

## Background

Pyrimidine derivatives are found to have a wide range of chemotherapeutic effects including angiogenic [1], enzyme inhibitory effects [2, 3] and anti-leshiminal activity [4]. They have also been used as analgesics and anti-parkinsonian agents [5, 6], as modulators of TRPV1 (Transient Receptor Potential Vanilloid Receptor 1) [7], as anticancer agents [8–10], as pesticides [11], as phosphate inhibitors [12, 13], for treatment of circulatory system diseases [14]. They are also known to have antimicrobial [15–17], anti-inflammatory [18], and anti-insecticidal [19] properties in addition to acetyl cholinesterase inhibitory activity [20]. Thiazolopyrimidine and thiazolo-pyrimidopyrimidine compounds have attracted our interest due to the wide range of biological activities they exhibit. For instance, thiazolopyrimidines are known to exhibit hypoglycemic, hypolipidemic, antidiabetic [21] and antibacterial and anti-tubercular activities [22]. The microwave technique has many benefits over conventional synthetic methods. Reduction of reaction times, minimization energy consumption, management of analytical waste, improving yields and increasing safety for the operator were the main benefits of this technique [23–28]. The use of microwave depends on the ability of the reacting molecules to efficiently absorb microwave energy taking advantage of microwave dielectric heating phenomena such as dipolar polarization or ionic conduction mechanisms. This leads to rapid internal heating (in-core volumetric heating) by direct interaction of electromagnetic radiation with the reacting molecules. Even though diverse types of microwave reactors and processing options are available currently, most of the microwave synthetic protocols have been reported in sealed reactors [29]. The rapid heating and high temperatures resulting in microwave chemistry makes it obvious based on the application of the Arrhenius equation, [*k* = *A* exp(− *E*_a_/R*T*)] that transformations that reach completion in hours under conventional heating in a solvent, would be completed in only minutes using superheated solvents under microwave conditions using a autoclave type sealed reactor. In addition the rapid heating generally produced in microwave chemistry may sometimes lead to altered product distributions as compared to reactions conducted under conventional heating if the product distribution is determined by complex temperature dependent kinetics [29, 30]. This may be the reason why in many instances reactions performed under microwave irradiation at an optimized reaction temperature lead to lesser side products in comparison to reactions performed under conventional heating where the reaction temperature is often non-optimal [29–31]. Encouraged by the findings of the previously reported work [34–36] we herein report the use of microwave-assisted technique for preparing new derivatives of a series of thiazolopyrimidine and thiazolopyrimidothiazolopyrimidine for evaluation of their antimicrobial activity. The absorption and fluorescence emission of some of the prepared compounds were studied in dioxane, revealing that the substituents altered both the absorption and fluorescence emission maxima.

## Results and discussion

### Chemical characterization

**Scheme 1:**
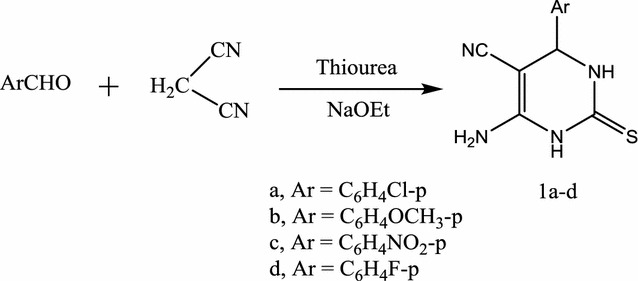
Synthesis of pyrimidine-5-carbonitriles **1a**–**d**

The above discussed medicinal and biological properties of fused pyrimidine derivatives, prompted us to carry out the synthesis of a series of new thiazolopyrimidine and thiazolodipyrimidine derivatives using microwave chemistry in conjunction with conventional chemical synthesis. The reaction of bifunctional reagents with 6-amino-4-aryl-2-thioxo-1,2,3,4-tetrahydropyrimidine-5-carbonitrile derivatives **1a**–**d**, afforded a simple and efficient approach for the synthesis of the target molecules. The synthesized target molecules were evaluated for their antimicrobial activity. The starting materials **1a**–**d** were obtained by the one pot reaction of aromatic aldehydes, malononitrile and thiourea in an alcoholic sodium ethoxide solution (Scheme 1). Compounds **1a**–**d** were characterized using elemental analysis as well as spectroscopic data. Compounds **1a**, **b** were prepared according to literature procedures [33, 40].

The IR (ʋ, cm^−1^) spectra of **1a**–**d** showed absorption bands at 3350, 3270 and 3180 (NH, NH_2_), 3050, 2980 (CH), 2217 (CN). ^1^H-NMR (DMSO-d6) of **1d**, as an example, showed signals δ (ppm) at 5.02 (s, 1H, pyrimidine H-4), 6.65 (s, 2H, NH_2_, D_2_O exchangeable), 7.23 (d, 2H, J = 7.8 HZ, aromatic protons), 7.51 (d, 2H, J = 7.8 HZ, aromatic protons), 8.65 (s, 1H, NH, D_2_O exchangeable) and 9.53 (s, 1H, NH, D_2_O exchangeable). Its ^13^C-NMR (DMSO-d6) showed signals δ (ppm) at 54.5 (pyrimidine C-4), 62.3 (pyrimidine C-5), 112.2, 117.1, 127.1, 133.2, 141.2, 160.5 (aromatic carbons + CN and pyrimidine C-6) and 175.3 (C=S). Mass spectrum of **1d**, as an example, showed the molecular ion Peak at *m/z* 247 (8.5%) corresponding to the molecular formula C_11_H_9_N_4_FS (“Experimental”).

**Scheme 2:**
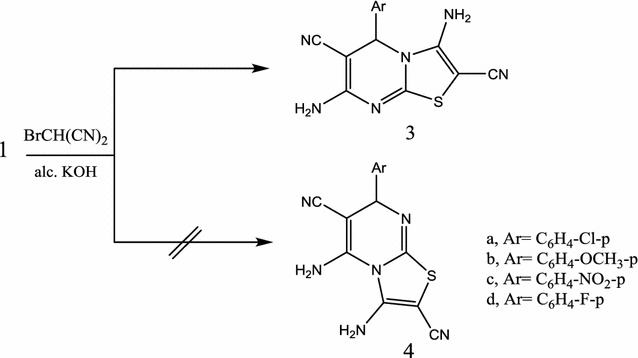
Synthesis of thiazolopyrimidine **3**

Each of **1a**–**d** reacted with equimolar amount of monobromomalononitrile (**2**), in ethanolic potassium hydroxide solution, yielded in each case a single product which could be formulated to be either 5*H*-thiazolo[3,2-*a*]pyrimidine structure **3** or its isomeric structure 7*H*-thiazolo[3,2-*a*]pyrimidine **4** (Scheme 2). Preferring structure **3** over **4** was based on the comparison of the ^1^H-NMR spectral data for compounds **1** and **3**. Thus, the ^1^H-NMR spectrum of **3b** as an example revealed, in addition to the methoxy group, aromatic and NH_2_ proton signals, a singlet (1H) at δ 6.41 assigned to the pyrimidine H-5. The downfield for the pyrimidine H-5 in **3b** compared with the pyrimidine H-4 in **1b**, which appeared at δ = 5.12 ppm, indicates that the moiety nearby H-5 in **3b** differs from that of H-4 in **1b**. Therefore, structure **3** could be initially assigned for the reaction products.

**Scheme 3:**
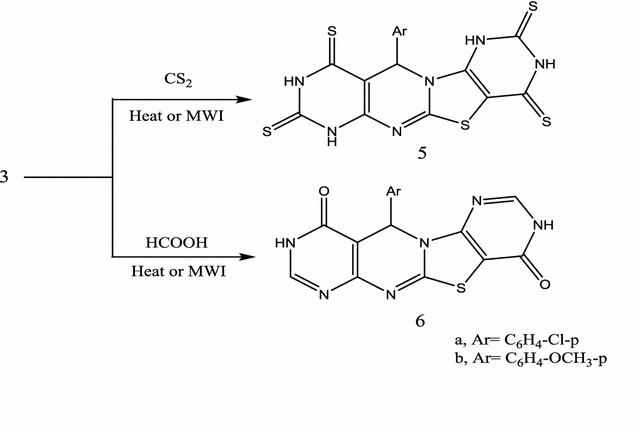
Synthesis of pyrimidothiazolopyrimidopyrimidine **5**, **6**

The IR (ʋ, cm^−1^) spectra of **3a**–**d** displayed absorption bands characterized for 2NH_2_ and 2CN groups. ^1^H-NMR (DMSO-d6) for compound **3b**, as an example, showed signals δ (ppm) at 6.53, 6.95 characterized 2NH_2_ (D_2_O exchangeable) groups. Its ^13^C-NMR (DMSO-d6) showed signals δ (ppm) at 52.5 (pyrimidine C-5), 56.7 (OCH_3_), 60.3 (thiazole C-2), 81.2 (pyrimidine C-6), 113.2, 117.1 (2CN), 125.1, 129.3, 135.2, 145.2, 159.5, 160.2 (aromatic carbons + C-8a and thiazole C-3) and 165.3 (pyrimidine C-7). Its mass spectrum showed the molecular ion Peak at *m/z* 324 (11.4%) corresponding to the molecular formula C_15_H_12_N_6_OS. Compounds (**3a**–**d**) gave compatible elemental and spectral data (“Experimental”). Comparing compounds formed by the traditional method and those prepared by the microwave assisted conditions indicates reduction of the reaction time to 8 min instead of 24 h standing. Also, the reaction yields were increased from 42–55 to 69–88%. Compound **3**, as typical dienaminonitriles, allowed for hetero-annelations performing access to fused pyrimidines. They could be used as precursors for the preparation of pyrimidothiazolopyrimidopyrimidines. Thus, a mixture of each of **3a**, **b** were heated under reflux with an excess of carbon disulphide to yield, in each case, the corresponding 11-aryl-11*H*-1,2,3,4,7,8,9,10-octahydropyrimido[4″,5″:4′,5′]thiazolo[3′,2′-*a*]pyrimido[4,5-*d*]pyrimidine-2,4,8,10-tetrathione **5a**, **b** (Scheme 3).

**Scheme 4:**
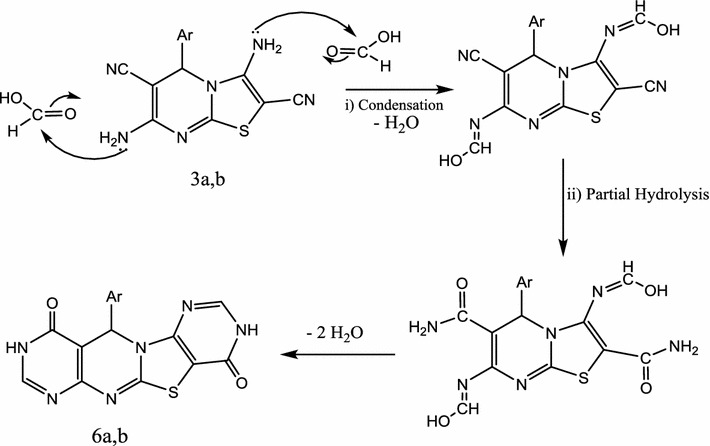
Mechanism for the formation of **6**

Finally, treatment of **3a**, **b** with formic acid by heating several hours yielded 11-aryl-9*H*-1,3,6,7-tetrahydropyrimido-[5″,4″:4′,5′]thiazolo[3′,2′-*a*]pyrimido[4,5-*d*]pyrimidine-4,10-dione **6a**, **b** (Scheme 3). Compounds **5**, **6** gave compatible elemental and spectral data (“Experimental”). Formation of **6** is assumed to proceed via condensation reaction followed by partial hydrolysis and finally removal of two molecules of water (Scheme 4).

**Scheme 5:**
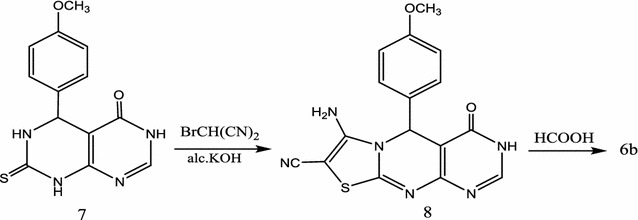
Synthesis of **6b** in step wise sequence

Compound **6b** could be synthesized in step wise sequence by heating 5-(4-methoxyphenyl) 7-thioxo-5,6,7,8-tetrahydro-3*H*-pyrimido[4,5-d]pyrimidin-4-one (**7**) [33] with bromomalononitrile (**2**) in ethanolic potassium hydroxide solution to produce 7-amino-5-(4-methoxyphenyl)-4-oxo-3,5-dihydro-4*H*-pyrimido[4,5-d]thiazolo[3,2-a]pyrimidine-8-carbonitrile (**8**). Compound **8** gave compatible elemental and spectral data (“Experimental”). On boiling under reflux compound **8** with formic acid for several hours yielded the desired compound. The obtained product was identical in all aspects (m.p., mixed m.p., IR spectra) to product **6b** (Scheme 5).

**Scheme 6:**
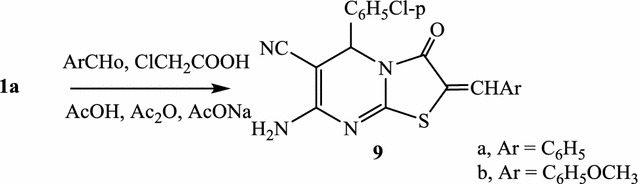
Formation of 2-arylmethylene-7-amino-5-(4-chloropenyl)-3-oxo-2,3-dihydro-5-*H*-thiazolo[3,2-a]pyrimidine-6-carbonitrile

**Scheme 7:**
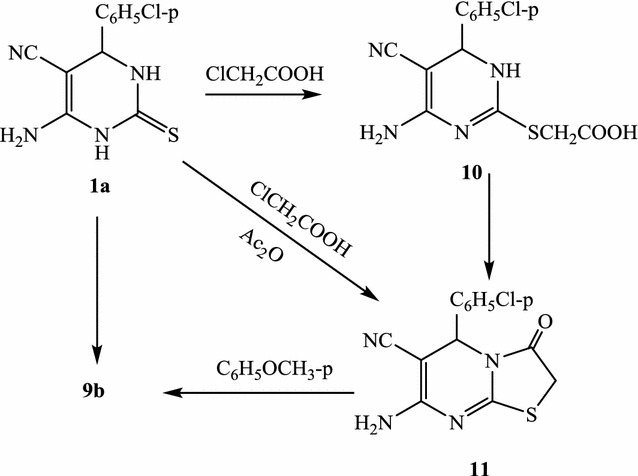
Supporting of structure **9**

As an extension of alkylation and cycloalkylation, compound **1a** was heated under reflux with a mixture of chloroacetic acid, aromatic aldehyde and anhydrous sodium acetate in acetic acid/acetic anhydride solution to give 2-arylmethylene-7-amino-5-(4-chloropenyl)-3-oxo-2,3-dihydro-5-*H*-thiazolo[3,2-a]pyrimidine-6-carbonitrile (**9a**, **b**), in good yields (Scheme 6). IR (ύ, cm^−1^) spectra of **9** display absorption bands around 3350 and 3240 (NH_2_), 2213 (CN) and 1695 (CO). 1H-NMR spectrum (DMSO-d6) of **9b**, as an example, shows signals at δ 3.41 ppm (s, 3H, CH_3_), 5.07(s, 1H, pyrimidine H-5), 7.20–7.67 (m, 8H, aromatic protons + 1H, methine proton) and 8.3 (s, 2H, NH_2_, D2O exchangeable). Mass spectrum of **9b**, as an example, gives the molecular ion peaks at *m/z* 422 (35.4%), 424 (12.2%) and the base peak at *m*/*z 302*. In support of structure **9**, compound **9b**, as an example, could be synthesized step wisely. Thus, when compound **1a** was heated under reflux with chloroacetic acid and sodium acetate in acetic acid, it gave the 2-carboxymethylthio derivative **10**. The latter compound could be cyclized by heating with acetic acid/acetic anhydride at 100 °C to give 7-amino-5-(4-chloropenyl)-3-oxo-2,3-dihydro-5-*H*-thiazolo[3,2-a]pyrimidine-6-carbonitrile (**11**). Upon heating under reflux **11** with *p*-methoxybenzaldehyde in acetic acid, in presence of anhydrous sodium acetate, **9b** was obtained (Scheme 7). Compounds **10** and **11** gave the expected values in elemental analyses and spectral data (“Experimental”).

**Fig. 1:**
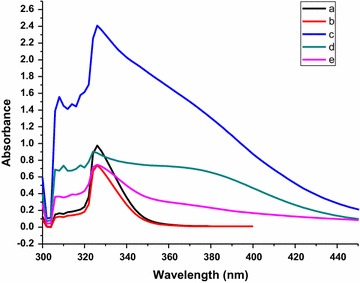
UV–Vis absorption spectra of the prepared compounds: a = **1a**, **3a**, **6a**; b = **5a**, **6a**, **6b**, **9b**; c = **3b**, **3d**, **10**; d = **1b**, **1d**; e = **1c**, **3c**, **5b**, **9a**

We have recently been attentive in carrying out synthesis of some heterocyclic compounds, with expected biological activity, under environmentally friendly, time saving microwave-assisted conditions [34–39]. Accordingly, we resynthesized the previously described compounds **1a**–**d**, **3a**–**d**, **5a**, **b** and **6a**, **b** under microwave conditions, aiming to increase reaction yields and reduce the reaction times, the difference in the outcome of the MW-assisted and thermal reactions are shown in Table 1. The outcomes of these preparations indicated that reaction yields were improved by 17–23% compared to the conventional methods. Also reaction times were considerably reduced. Figure 1 summarizes the outcome of using microwave technique for the preparation of the abovementioned compounds.

**Table 1:** The difference in the outcome of the MW-assisted and thermal reactions for the synthesis of compounds **1a**–**d**, **3a**–**d**, **5a**, **b** and **6a**, **b**

Compound no.	Reaction yield %		Reaction time min	
	Microwave	Conventional method	Microwave	Conventional method
**1a**	88	45	10	1440
**1b**	74	43	14	1440
**1c**	87	52	9	1440
**1d**	83	45	10	1440
**3a**	82	60	8	1440
**3b**	76	50	5	1440
**3c**	74	43	5	1440
**3d**	74	43	7	1440
**5a**	82	55	8	480
**5b**	76	43	8	480
**6a**	72	39	8	600
**6b**	70	39	8	600

### Biological evaluation

#### Antimicrobial evaluation

The newly prepared compounds were verified for their antimicrobial action against different microorganisms such as: *Escherichia coli*, *Pseudomonas putida*, *Bacillus subtilis*, *Streptococcus lactis*, *Aspergillus niger*, *Penicillium* sp. and *Candida albicans*. The initial screening of the investigated compounds was achieved using the filter paper disc-diffusion method. Compounds **1a**, **b**, **3a**, **b**, **5a**, **6a**, **8**, **9a** and **10** showed moderate to slight inhibitory action towards the microorganisms. Other compounds showed slight to no sensitivity at all to the mentioned organisms, the results are listed in Table 2.

**Table 2:** Antimicrobial activities of the newly synthesized compounds

Compound no.	IZ^*^ ± SD^**^						
	Gram-negative		Gram-positive		Fungi		Yeast
	*E. coli*	*P. putida*	*B. subtilis*	*S. lactis*	*A. niger*	*P.* sp.	*C. albicans*
**1a**	14 ± 0.58	10 ± 0.20	7 ± 0.29	8 ± 0.50	5 ± 0.29	5 ± 0.15	0
**1b**	12 ± 0.50	9 ± 0.15	6 ± 0.58	7 ± 0.29	4 ± 0.12	2 ± 0.20	0
**1c**	6 ± 0.29	3 ± 0.29	0	0	2 ± 0.15	2 ± 0.12	0
**1d**	3 ± 0.15	2 ± 0.20	0	0	0	0	0
**3a**	15 ± 0.58	11 ± 0.12	9 ± 0.50	6 ± 0.15	7 ± 0.58	5 ± 0.12	0
**3b**	12 ± 0.15	7 ± 0.29	7 ± 0.12	5 ± 0.50	7 ± 0.20	5 ± 0.50	0
**3c**	0	0	0	0	0	0	0
**3d**	3 ± 0.29	2 ± 0.20	0	0	0	0	0
**5a**	1 ± 0.20	0	7 ± 0.29	8 ± 0.29	6 ± 0.20	4 ± 0.29	3 ± 0.29
**5b**	1 ± 0.12	3 ± 0.15	7 ± 0.58	8 ± 0.20	6 ± 0.12	5 ± 0.15	3 ± 0.20
**6a**	1 ± 0.29	0	6 ± 0.12	8 ± 0.15	6 ± 0.20	4 ± 0.15	3 ± 0.15
**6b**	1 ± 0.12	2 ± 0.20	7 ± 0.20	8 ± 0.20	6 ± 0.15	4 ± 0.29	4 ± 0.12
**8**	14 ± 0.58	12 ± 0.50	7 ± 0.50	7 ± 0.12	6 ± 0.58	5 ± 0.29	2 ± 0.29
**9a**	15 ± 0.58	13 ± 0.15	8 ± 0.15	7 ± 0.29	7 ± 0.50	4 ± 0.12	2 ± 0.50
**9b**	10 ± 0.15	7 ± 0.29	6 ± 0.20	5 ± 0.29	2 ± 0.12	2 ± 0.20	0
**10**	16 ± 0.76	12 ± 0.15	9 ± 0.12	8 ± 0.58	5 ± 0.29	4 ± 0.29	3 ± 0.12
**11**	6 ± 0.20	3 ± 0.15	2 ± 0.29	0	2 ± 0.12	0	0
Chloramphenicol	22	21	18	19	1	0	0
Ampicillin	24	20	19	22	0	0	0

^*^inhibition diameter zones expressed in millimeters (mm); ^**^ standard deviation; *E. coli*, *Escherichia coli*; *P. putida*, *Pseudomonas putida*; *B. subtilis*, *Bacillus subtilis*; *S. lactis*, *Streptococcus lactis*; *A. niger*, *Aspergillus niger*; *P.* sp., *Penicillium* sp.; *C. albicans*, *Candida albicans*

The sensitivity of microorganisms to the tested compounds is identified in the following manner: highly sensitive = inhibition zone: 15–20 mm; moderately sensitive = inhibition zone: 10–15 mm; slightly sensitive = inhibition zone: 1–10 mm; not sensitive = inhibition zone: 0 mm; each result represents the average of triplicate readings

### Fluorescence and absorption spectra

The UV–Vis absorption spectra of all compounds as well as the fluorescence spectra of the compounds exhibiting fluorescence in solution were measured in 1,4-dioxane. It is clear from Fig. 1 that the prepared compounds exhibit UV–Vis absorption spectra in the region of 250–500 nm with a maximum absorption at 326 nm. The difference in the intensity of the prepared compounds depends on the difference of their chemical structures. The probabilities of compounds towards excitation from the ground state to the singlet excited state (absorption cross-section σ_a_) by absorbing photons at wavelength of 326 nm were calculated using Eq. (1) as follows [40]: σ_a_ = 0.385 × 10^−20^ ε where: the molar absorptivity ε was calculated from Beer–Lambert law Eq. (2):

$${\text{A}} = \log {\text{I}}_{0} /{\text{I}} =\upvarepsilon{\text{C L}}$$

where: A: absorbance, I_0_ and I: intensities of incident and emerged light from the sample, C: molar concentration of compounds and L is the light path (1 cm).

The absorption and emission spectral maxima are listed in Table 3. The fluorescence properties of the compounds depend on the presence of electron-with donating and electron-withdrawing substituents on the acceptor part. The acceptor part of 2-carboxymethylthio derivative **10** contains carboxyl group when compared with other compounds. Hence, due to the less positive inductive effect of **10**, the donating tendency becomes less and compound **10** exhibits high quantum yield φ_*f*_ of 0.73, much higher than other compounds.

**Table 3:** Absorption (λ_A_), fluorescence (λ_F_) maxima (nm) and quantum yield φ_f_ (%) of the prepared compounds

Compound	λ_A_	λ_F_	ε, I/Mcm × 10^4^	σ_a_ (10^−16^) cm^2^	Quantum yield (%) φ_f_
**1a**	321	–	2.8	1.07	–
**1b**	317	–	2.9	1.11	–
**1c**	299	–	3.1	1.19	–
**1d**	309	–	2.9	1.11	–
**3a**	320	372	3.2	1.23	0.68
**3b**	325	–	3.3	1.27	–
**3c**	315	–	3.1	1.19	–
**3d**	325	–	3.0	1.15	–
**5a**	326	–	3.4	1.3	0.63
**5b**	295	–	5.8	2.23	–
**6a**	320	360	2.3	1.07	–
**6b**	325	–	6.2	2.38	–
**9a**	315	–	5.5	2.11	–
**9b**	322	–	6.5	2.5	–
**10**	326	537	7.8	3	0.73

**Fig. 2:**
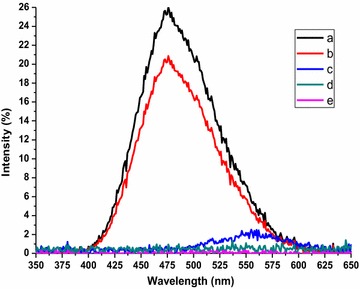
Emission spectra of the prepared compounds: a = **10**; b = **3a**; c = **5a**; d = **1a**–**d**, **2a**–**d**, **3b**, **3c**, **3d**; e = **6**–**9**

No fluorescence was detected in solution for all studied compounds except **10**, **3a** and **5a** (Table 3; Figs. 1, 2). Compounds **3a** and **5a** exhibited intense fluorescence while compounds **10** exhibited high quantum yield φ_*f*_ of 0.68 and 0.63 and 0.73 respectively, which may be due to the presence a polycyclic compounds with tetrathione moiety and electron-withdrawing substituents, enabling extended conjugation (Table 3). Simultaneously, it was observed that only compound **10** showed fluorescence in both solution and solid phase, and the fluorescence maximum in solid phase was shifted bathochromically by about 50 nm compared with the maximum in solution. Conversely, compounds **3a** and **5a** exhibited fluorescence only in solution.

## Experimental

### General

A Gallenkamp melting point apparatus was used to determine melting points and IR spectra (KBr discs) were recorded on a Shimadzu FTIR-8201PC Spectrophotometer. ^1^H-NMR and ^13^C-NMR spectra were verified on a Varian Mercury 300 MHz and a Varian Gemini 200 MHz spectrometers using TMS as an internal standard and DMSO-*d6* as a solvent and the chemical shifts were expressed as δ (ppm) units. Shimadzu GCMS-QP1000EX instrument were used to record Mass spectra using an inlet type sample injection at 70 eV. The Microanalytical Center of Cairo University performed the microanalyses. Microwave reactions were performed with a Millstone Organic Synthesis Unit (Micro SYNTH with touch control terminal) with a continuous focused microwave power delivery system in a pressure glass vessel (10 mL) sealed with a septum under magnetic stirring. A calibrated infrared temperature control was used to monitor the temperature of the reaction mixture under the reaction vessel with a pressure sensor connected to the septum of the vessel to control the pressure. Ultraviolet–visible absorption spectra were measured on a PerkinElmer Lambda 35 Spectrophotometer at room temperature. Steady-state fluorescence spectra were measured on a PerkinElmer LS 55 spectrophotometer. The prepared compounds were dissolved in precleaned amber glass vials (1-cm cell) containing dioxane as solvent in concentration of 1 × 10^−5^ M (King Khalid University).

Compounds **1a**, **b** were prepared according to literature procedures [33, 41].

#### 6-Amino-4-aryl-2-thioxo-1,2,3,4-tetrahydro-pyrimidine-5-carbonitriles **1a**–**d**

*Method A* A solution of thiourea (0.76 g, 0.01 mol), malononitrile (0.66 g, 0.01 mol) and the appropriate aromatic aldehyde in sodium ethoxide (sodium metal 0.23 g, 0.01 mol in absolute ethanol 30 mL) was stirred at room temperature for 24 h. Then the mixture was poured onto ice-cold water and neutralized by dilute HCl. The solid precipitate so-formed was filtered off, washed with water and crystallized from ethanol.

*Method B* The same reactants of method A in 5 mL sodium ethoxide solution were heated in microwave oven at 500 W and 140 °C for about 10 min. Compounds **1a**–**d** was produced by treating the reaction mixture in a similar manner of method A.

6-Amino-4-(4-chlorophenyl)-2-thioxo-1,2,3,4-tetrahydro-pyrimidine-5-carbonitrile (**1a**). The aromatic aldehyde used was 4-chlorobenzaldehyde (1.40 g, 0.01 mol), (yield 88%, 2.32 g) according to method B. Compound **1a** was obtained as yellow crystals, yield for method A, 52%, m.p. 121–123 °C. ^1^H-NMR: δ (ppm) 1.52 (s, 1H, –SH), 3.41 (s, 1H, NH, D_2_O exchangeable), 4.31 (s, 2H, NH_2_, D_2_O exchangeable), 4.81 (s, 1H, –CH), 6.87–7.23 (m, 4H, Ar–H). ^13^C-NMR: δ (ppm) 45.8 (pyrimidine C-4), 68.2 (pyrimidine C-5), 117.2 (CN), 126.5, 127.6, 128.4, 129.0, 133.1, 158.3 (aromatic carbons + pyrimidine C-6) and 170.1 (C=S). IR (KBr) ʋ: 3370, 3252 and 3180 (NH + NH_2_), 3050, 2950 (CH), 2215 (CN), 1640, 1543 cm^−1^ (Aromatic C=C). MS (70 eV): (M+2) *m/z* 266 (5.8%), (M+) 264 (18.6%). Anal. Calcd. For C_11_H_9_N_4_SCl (264.5): C (49.91%), H (3.43%), N (21.16%), S (12.12), Cl (13.44%); Found: C (49.85%), H (3.38%), N (20.87%), S (11.87%), Cl (13.38).

6-Amino-4-(4-methoxyphenyl)-2-thioxo-1,2,3,4-tetrahydro-pyrimidine-5-carbonitrile (**1b**). The aromatic aldehyde used was 4-methoxybenzaldehyde (1.22 mL, 0.01 mol), (yield 74%, 1.92 g) according to method B. Compound **1b** was obtained as fine yellow crystals, yield for method A, 48%, m.p. 120–122 °C. ^1^H-NMR: δ (ppm) 1.45 (s, 1H, –SH), 3.41 (s, 1H, NH, D_2_O exchangeable), 3.86 (s, 3H, –OCH_3_), 4.40 (s, 2H, NH_2_, D_2_O exchangeable), 4.67 (s, 1H, –CH), 6.76–7.12 (m, 4H, Ar–H). ^13^C-NMR: δ (ppm) 46.2 (–OCH_3_), 52.3 (pyrimidine C-4), 60.0 (pyrimidine C-5), 117.3 (CN), 126.5, 127.6, 128.4, 129.0, 133.1, 158.3 (aromatic carbons + pyrimidine C-6) and 168.4 (C=S). IR (KBr) ʋ: 3377, 3260 and 3180 (NH + NH_2_), 3050, 2925 (CH), 2213 (CN), 1645, 1543 cm^−1^ (Aromatic C=C). MS (70 eV): (M+) *m/z* 260 (13.5%). Anal. Calcd. For C_12_H_12_N_4_OS (260.32): C (55.37%), H (4.65%), N (21.52%), S (12.30); Found: C (55.31%), H (4.59%), N (21.10%), S (11.87%).

6-Amino-4-(4-nitrophenyl)-2-thioxo-1,2,3,4-tetrahydro-pyrimidine-5-carbonitrile (**1c**). The aromatic aldehyde used was 4-nitrobenzaldehyde 1.51 g (0.01 mol). Compound 1c was obtained as fine yellow crystals, yield for method A, 52%, 1.43 g and for method B, 87%, 2.39 g), m.p. 200–203 °C. ^1^H-NMR: δ (ppm) 4.95 (s, 1H, pyrimidine H-4), 6.61(s, 2H, NH_2_, D_2_O exchangeable), 7.48 (d, 2H, Ar–H, *J *=* 7.4* Hz), 7.82 (d, 2H, Ar–H, *J *=* 7.4* Hz), 8.84 (s, 1H, NH, D_2_O exchangeable) and 9.53 (s, 1H, NH, D_2_O exchangeable). ^13^C-NMR: δ (ppm) 53.5 (pyrimidine C-4), 62.2 (pyrimidine C-5), 117.2 (CN), 124.5, 127.6, 144.4, 150.0, 168.1 (aromatic carbons + pyrimidine C-6) and 173.1 (C=S). IR (KBr) ʋ: 3350, 3270 and 3180 (NH + NH_2_), 3050, 2980 (CH), 2217 (CN), 1605, 1500 cm^−1^ (Aromatic C=C). MS (70 eV): (M+) *m/z* 275 (16.2%). Anal. Calcd. for C_11_H_9_N_5_O_2_S (275.25): C (47.99%), H (3.29%), N (25.44%), S (11.64); Found: C (47.83%), H (3.14%), N (25.35%), S (11.53%).

6-Amino-4-(4-florophenyl)-2-thioxo-1,2,3,4-tetrahydro-pyrimidine-5-carbonitrile (**1d**). The aromatic aldehyde used was 4-flourobenzaldehyde, 1.07 mL (0.01 mol). Compound **1d** was obtained as yellow crystals, yield, for method A, 45%, 1.11 g and for method B 83%, 2.05 g) m.p. 246–247 °C. ^1^H-NMR: δ (ppm) 5.02 (s, 1H, pyrimidine H-4), 6.65 (s, 2H, NH_2_, D_2_O exchangeable), 7.23 (d, 2H, J = 7.8 HZ, aromatic protons), 7.51 (d, 2H, J = 7.8 HZ, aromatic protons), 8.65 (s, 1H, NH, D_2_O exchangeable) and 9.53 (s, 1H, NH, D_2_O exchangeable). ^13^C-NMR: δ (ppm) 54.5 (pyrimidine C-4), 62.3 (pyrimidine C-5), 112.2, 117.1, 127.1, 133.2, 141.2, 160.5 (aromatic carbons + CN and pyrimidine C-6) and 175.3 (C=S). IR (KBr) ʋ: 3300, 3220 and 3140 (NH + NH_2_), 3050, 2980 (CH), 2217 (CN), 1605, 1500 cm^−1^ (Aromatic C=C). MS (70 eV): (M+) *m/z* 248 (11.2%). Anal. Calcd. For C_11_H_9_N_4_SF (248.24): C (53.21%), H (3.64%), N (22.56%), S (12.91), F (7.64); Found: C (53.24%), H (3.53%), N (22.38%), S (12.51%), F (6.97%).

#### 3,7-Diamino-5-aryl-5*H*-thiazolo[3,2-a]pyrimidine-2,6-dicarbonitriles (**3a**–**d**)

*Method A* To a warm ethanolic potassium hydroxide solution [prepared by dissolving KOH (0.56 g, 0.01 mol) in ethanol (50 mL)] of each of **1a**–**d** [(1a, 2.64 g; 1b, 2.60 g; 1c, 2.75 g; 1d, 2.48 g; 0.01 mol)],bromomalononitrile (**2**) (1.45 g, 0.01 mol) was added portion-wise and stirred at room temperature for 24 h. Whereby the solid product that separated upon dilution with water was filtered off and crystallized from the proper solvent.

*Method B* The same reactants of method A in 5 mL ethanolic potassium hydroxide solution were heated in microwave oven at 500 W and 140 °C for 5–8 min. compounds **3a**–**d** was produced by treating the reaction mixture in a similar manner of method A.

3,7-Diamino-5-(4-chlorophenyl)-5*H*-thiazolo[3,2-a]pyrimidine-2,6-dicarbonitriles (**3a**) was crystallized from dil. dioxane as brown crystals, yield for method A, 60%, 1.96 g and for method B, 82%, 2.68 g) m.p. 220–222 °C. ^1^H-NMR: δ (ppm) 6.10 (s, 1H, pyrimidine H-5), 6.83 (s, 2H, NH_2_, D_2_O exchangeable), 7.24 (s, 2H, NH_2_, D_2_O exchangeable), 7.73 (d, 2H, *J *= 7.4 HZ, aromatic protons) and 7.95 (d, 2H, *J *= 7.4 HZ, aromatic protons). ^13^C-NMR: δ (ppm) 55.6 (pyrimidine C-5), 59.3 (thiazole C-2), 81.1 (pyrimidine C-6), 113.9, 117.3 (2CN), 127.1, 129.4, 133.2, 141.5, 158.8, 159.3 (aromatic carbons + C-8a and thiazole C-3) and 167.2 (C-7). IR (KBr) ʋ: 3310, 3240 (NH_2_), 3030, 2984 (CH) and 2217, 2219 (2CN). MS (70 eV): (M+2) *m/z* 330 (2.8%), (M+) 328 (9.4%). Anal. Calcd. For C_14_H_9_N_6_SCl (328.75): C (51.14%), H (2.75%), N (25.56%), S (9.75), Cl (10.78); Found: C (51.10%), H (2.56%), N (25.14%), S (9.51%), Cl (10.21%).

3,7-Diamino-5-(4-methoxyphenyl)-5*H*-thiazolo[3,2-a]pyrimidine-2,6-dicarbonitriles (**3b**) was crystallized from ethanol as beige crystals, yield for method A 50%, 1.62 g and for method B 76%, 2.46 g) m.p. 224–226 °C. ^1^H-NMR: δ (ppm) 3.85 (s, 3H, OCH_3_), 6.41 (s, 1H, pyrimidine H-5), 6.63 (s, 2H, NH_2_, D_2_O exchangeable), 7.12 (s, 2H, NH_2_, D_2_O exchangeable), 7.75 (d, 2H, *J *= 7.3 HZ, aromatic protons) and 7.95 (d, 2H, *J *= 7.3 HZ, aromatic protons). ^13^C-NMR: δ (ppm) 52.5 (pyrimidine C-5), 56.7 (OCH_3_), 60.3 (thiazole C-2), 81.2 (pyrimidine C-6), 113.2, 117.1 (2CN), 125.1, 129.3, 135.2, 145.2, 159.5, 160.2 (aromatic carbons + C-8a and thiazole C-3) and 165.3 (pyrimidine C-7). IR (KBr) ʋ: 3310, 3240 (NH_2_), 3030, 2984 (CH) and 2217, 2220 (2CN). MS (70 eV): (M+) *m/z* 324 (10.4%). Anal. Calcd. For C_15_H_12_N_6_OS (324.31): C (55.54%), H (3.70%), N (25.91%), S (9.88); Found: C (55.31%), H (3.63%), N (25.14%), S (9.53%).

3,7-Diamino-5-(4-nitrophenyl)-5*H*-thiazolo[3,2-a]pyrimidine-2,6-dicarbonitriles (**3c**) was crystallized from ethanol as brown crystals, yield for method A, 43%, 1.45 g and for method B, 74%, 2.50 g) m.p. 243–245 °C. ^1^H-NMR: δ (ppm) 5.84 (s, 1H, pyrimidine H-5), 6.56 (s, 2H, NH_2_, D_2_O exchangeable), 6.81 (s, 2H, NH_2_, D_2_O exchangeable), 7.75 (d, 2H, *J *= 7.4 HZ, aromatic protons) and 8.35 (d, 2H, *J *= 7.3 HZ, aromatic protons). 13C-NMR: δ (ppm) 52.5 (pyrimidine C-5), 59.3 (thiazole C-2), 82.3 (pyrimidine C-6), 115.2, 118.3 (2CN), 125.3, 129.4, 135.2, 144.2, 159.5, 160.5 (aromatic carbons + C-8a and C-3) and 164.7 (pyrimidine C-7). IR (KBr) ʋ: 3310, 3240 (NH_2_), 3035, 2985 (CH) and 2218, 2223 (2CN). MS (70 eV): (M+) *m/z* 339 (7.8%). Anal. Calcd. For C_14_H_9_N_7_O_2_S (339.29): C (49.55%), H (2.67%), N (28.89%), S (9.45); Found: C (49.35%), H (2.60%), N (28.16%), S (9.11%).

3,7-Diamino-5-(4-florophenyl)-5*H*-thiazolo[3,2-a]pyrimidine-2,6-dicarbonitriles (**3d**) was crystallized from dioxane as brown crystals, yield for method A, 43%, 1.34 g and for method B, 74%, 2.30 g) m.p. 251–253 °C. ^1^H-NMR: δ (ppm) 5.91 (s,1H, pyrimidine H-5), 6.63 (s, 2H, NH_2_, D_2_O exchangeable), 6.22 (s, 2H, NH_2_, D_2_O exchangeable), 7.55 (d, 2H, *J *= 7.4 HZ, aromatic protons) and 7.84 (d, 2H, *J *= 7.4 HZ, aromatic protons). ^13^C-NMR: δ (ppm) 53.6 (pyrimidine C-5), 58.5 (thiazole C-2), 80.2 (pyrimidine C-6), 114.0, 117.3 (2CN), 127.1, 129.4, 133.2, 141.5, 158.8, 159.3 (aromatic carbons + C-8a and C-3) and 165.3 (C-7). IR (KBr) ʋ: 3310, 3240 (NH_2_), 3030, 2984 (CH) and 2217, 2219 (2CN). MS (70 eV): (M+) *m/z* 312 (4.6%). Anal. Calcd. For C_14_H_9_N_6_SF (312.29): C (53.84%), H (5.38%), N (26.90%), S (10.26), F (6.08); Found: C (53.75%), H (5.26%), N (26.27%), S (9.87%), F (5.77%).

#### 11-Aryl-11*H*-1,2,3,4,7,8,9,10-octahydropyrimido[4″,5″:4′,5′]thiazolo[3′,2′-a]pyrimido[4,5-d]pyrimidine-2,4,8,10-tetrathione **5a**, **b**

*Method A* Each of compounds **3a**, **b** (3a, 1.09 g, 3b, 1.08 g; 0.03 mol) was heated under reflux with an excess of carbon disulphide (20 mL) for 8 h. The reaction mixture was left to cool, the solid that precipitated was filtered off and crystallized from the proper solvent.

*Method B* Each of compounds **3a**, **b** (3a, 1.09 g, 3b, 1.08 g; 0.03 mol) in 6 mL carbon disulphide were heated in microwave oven at 500 W and 140 °C for 8 min. The reaction mixture was treated in a similar manner to method A to yield compounds **5a**, **b**.

11-(4-Chlorophenyl)-11*H*-1,2,3,4,7,8,9,10-octahydropyrimido[4″,5″:4′,5′]thiazolo[3′,2′-a]pyrimido[4,5-d]pyrimidine-2,4,8, 10-tetrathione (**5a**) was crystallized from dioxane as grey crystals, yield for method A, 55%, 0.88 g and for method B, 82%, 1.31 g, m.p. 248–250 °C. ^1^H-NMR: δ (ppm) 5.88 (s, 1H, pyrimidine H-10), 7.42–7.73 (m, 5H, Ar–H + NH, D_2_O exchangeable), 9.31 (s, 1H, NH, D_2_O exchangeable) and 12.85 (br, 2H, 2NH, D_2_O exchangeable). ^13^C-NMR: δ (ppm) 58.5 (pyrimidine C-10), 81.2 (C-4a), 110.2 (C-9a), 127.1, 132.2, 144.2, 156.3, 158.2, 166.7 (aromatic carbons + C-12a + C-5a + C-6a) and 171.3, 174.2, 188.4, 190.3 (4C=S). IR (KBr) ʋ: 3305, 3200 (NH), 3030, 2984 (CH), 1605, 1500 cm^−1^ (aromatic C=C). MS (70 eV): (M+2) *m/z* 483 (0.9%), (M+) 481 (3.2%). Anal. Calcd. For C_16_H_9_N_6_S_5_Cl (481.02): C (39.94%), H (1.88%), N (17.46%), S (33.32%), Cl (7.36%); Found: C (39.76%), H (1.78%), N (16.80%), S (32.86%), Cl (7.11%).

11-(4-Methoxyphenyl)-11*H*-1,2,3,4,7,8,9,10-octahydropyrimido[4″,5″:4′,5′] thiazolo-[3′,2′-a]pyrimido[4,5-d]pyrimidine-2,4,8,10-tetrathione (**5b**) was crystallized from dioxane as brown crystals, yield for method A, 43%, 0.68 g and for method B, 76%, 1.2 g, yield 43%, 2.04 g, m.p. 254–257 °C. ^1^H-NMR: δ (ppm) 3.42 (s, 3H, OCH_3_), 5.86 (s, 1H, pyrimidine H-10), 7.25–7.57 (m, 5H, Ar–H + NH, D_2_O exchangeable), 9.15 (s, 1H, NH, D_2_O exchangeable) and 12.24 (br, 2H, 2NH, D_2_O exchangeable). ^13^C-NMR: δ (ppm) 55.5 (pyrimidine C-10), 61.2 (OCH_3_), 79.4 (C-4a), 111.3 (C-9a), 125.1, 129.5, 143.5, 155.3, 158.2, 166.7 (aromatic carbons + C-12a + C-5a + C-6a) and 171.3, 173.4, 186.4, 188.6 (4C=S). IR (KBr) ʋ: 3305, 3200 (NH), 3030, 2984 (CH), 1605, 1500 cm^−1^ (aromatic C=C). MS (70 eV): (M+) *m/z* 476 (6.1%). Anal. Calcd. For C_17_H_12_N_6_OS_5_ (476.59): C (42.83%), H (2.53%), N (17.63%), S (33.64); Found: C (42.65%), H (2.50%), N (17.23%), S (33.22%).

#### 11-Aryl-9*H*-1,3,6,7-tetrahydropyrimido[5″,4″:4′,5′]thiazolo[3′,2′-a]pyrimido[4,5-d]pyri-midine-4,10-dione **6a**, **b**

*Method A* Each of compounds **3a**, **b** (3a, 1.09 g; 3b, 1.08 g; 0.03 mol) was heated under reflux with an formic acid (80%, 20 mL) for 10 h. The reaction mixture was left to cool and the solid that precipitated was filtered and crystallized from the proper solvent.

*Method B* Each of compounds **3a**, **b** (3a, 1.09 g; 3b, 1.08 g; 0.03 mol) in 5 mL formic acid (80%) were heated in microwave oven at 500 W and 140 °C for 8 min. compounds **6a**, **b** was obtained by treating the reaction mixture in a similar manner to method A.

11-(4-Chlorophenyl)-9*H*-1,3,6,7-tetrahydropyrimido[5″,4″:4′,5′]thiazolo[3′,2′-a]pyrimido[4,5-d]pyri-midine-4,10-dione **(6a**) was crystallized from dil. Dimethyl formamide as grey crystals, yield for method A, 39%, 0.49 g and for method B, 72%, 0.92 g, m.p. 272–275 °C. ^1^H-NMR: δ (ppm) 3.44 (s, 3H, OCH_3_), 4.89 (s, 1H, pyrimidine H-6), 7.31–7.25 (m, 4H, Ar–H), 8.13 (s, 1H, H-2), 8.34 (s, 1H, H-8) and 10.31 (s, 2H, 2NH, D_2_O exchangeable). ^13^C-NMR: δ (ppm) 58.5 (pyrimidine C-6), 119.4 (C-4a), 127.1, 129.2, 132.2, 138.3, 143.2, 147.3, 152.4 (aromatic carbons + C-6a + C-8 and C-12a), 154.4, 156.2 (C-5a + C-10a) and 162.4, 165.5 (2C=O). IR (KBr) ʋ: 3305, 3200 (NH), 3030, 2984 (CH), 1675 cm^−1^ (C=O). MS (70 eV): (M+2) *m/z 386* (3.2%), (M+) *m/z 384* (11.4%). Anal. Calcd. For C_16_H_9_N_6_O_2_SCl (384.75): C (49.94%), H (2.35%), N (21.84%), S (8.33%), Cl (9.21%); Found: C (49.82%), H (2.31%), N (21.53%), S (7.87%), Cl (8.75%).

11-(4-Methoxyphenyl)-9*H*-1,3,6,7-tetrahydropyrimido[5″,4″:4′,5′]thiazolo[3′,2′-a]pyrimido[4,5-d]pyri-midine-4,10-dione (**6b**) was crystallized from dil. Dimethyl formamide as grey crystals, yield for method A, 39%, 0.49 g and for method B, 70%, 0.88 g, m.p. 263–264 °C. ^1^H-NMR: δ (ppm) 3.8 (s, 3H, OCH_3_), 5.12 (s, 1H, pyrimidine H-6), 7.32–7.44 (m, 4H, Ar–H), 8.35 (s, 2H, H-2 + H-8) and 10.57 (s, 2H, 2NH, D_2_O exchangeable). ^13^C-NMR: δ (ppm) 56.6 (pyrimidine C-6), 62.3 (OCH_3_), 111.6 (C-4a), 120.4, 126.1, 133.3, 143.1, 147.5, 152.3 (aromatic carbons + C-6a + C-8 and C-12a), 154.4, 156.2 (C-5a + C-10a) and 162.4 165.5 (2C=O). IR (KBr) ʋ: 3305, 3200 (NH), 3030, 2984 (CH), 1675 cm^−1^ (C=O). MS (70 eV): (M+) *m/z* 380 (7.6%). Anal. Calcd. For C_17_H_12_N_6_O_3_S (380.31): C (53.68%), H (3.17%), N (22.09%), S (8.43%); Found: C (53.32%), H (2.89%), N (21.43%), S (7.87%).

#### 7-Amino-5-(4-methoxyphenyl)-4-oxo-3,5-dihydro-4*H*-pyrimido[4,5-d]thiazolo[3,2-a]pyrimidine-8-carbonitrile (**8**)

To a warm ethanolic potassium hydroxide solution of each of **7** (2.88 g, 0.01 mol), bromomalononitrile (**2**) (1.45 g, 0.01 mol) was added portion-wise with stirring. The reaction mixture was then left overnight at room temperature, whereby the solid product that separated upon dilution with water was filtered off and crystallized from ethanol as orange crystals, yield 22%, 0.77 g, m.p. 224–225 °C. ^1^H-NMR: δ (ppm) 3.6 (s, 3H, OCH_3_), 5.12 (s, 1H, pyrimidine H-5), 6.23 (s, 2H, NH_2_, D_2_O exchangeable), 7.15 (d, 2H, *J *= 7.1 HZ, aromatic protons) and 7.64 (d, 2H, *J *= 7.2 HZ, aromatic protons), 8.34 (s, 1H, H-2) and 10.55 (s, 1H, NH, D_2_O exchangeable). ^13^C-NMR: δ (ppm) 56.2 (OCH_3_), 59.0 (C-8), 60.1 (C-5), 113.5 (CN), 116.3, 123.3, 126.2, 132.6, 148.5 (aromatic carbons + C-4a + C-2 and C-10a), 156.7, 158.2 (C-9a + C-7) and 162.3 (C=O). IR (KBr) ʋ: 3280, 3220 and 3160 (NH + NH_2_), 3050, 2980 (CH), 2213 (CN), 1672 cm^−1^ (C=O). MS (70 eV): (M+) *m/z* 352 (9.3%). Anal. Calcd. For C_16_H_12_N_6_O_2_S (352.31): C (54.54%), H (3.42%), N (23.85%), S (9.10%); Found: C (54.11%), H (3.31%), N (23.53%), S (8.82%).

#### 2-Arylmethylene-7-amino-5-(4-chloropenyl)-3-oxo-2,3-dihydro-5-*H*-thiazolo[3,2-a]pyrimidine-6-carbonitrile (**9a**, **b**)

*Method A* A solution of **1a** (2.64 g; 0.01 mol) and chloroacetic acid (1.04 g, 0.01 mol), in a mixture of glacial acetic acid (20 mL) and acetic anhydride (20 mL) containing 1 g fused sodium acetate was heated under refluxed for 3 h with (0.01 mol) of each of benzaldehyde and *p*-methoxybenzaldehyde. The reaction mixture was poured onto water; the precipitated solid was filtered off, washed with water, dried and recrystallized from the proper solvent.

*Method B* A solution of 11 (2.73 g, 0.01 mol) in a mixture of glacial acetic acid (20 mL)/acetic anhydride (8 mL) containing 1 g fused sodium acetate was heated with *p*-methoxybenzaldehyde (1.49 g, 0.01 mol) under reflux for 1 h. The obtained solid was found to be identical in all aspects (IR, m.p., mixed m.p.) with compound **9b**.

7-Amino-5-(4-chloropenyl)-2-phenylylmethylene-3-oxo-2,3-dihydro-5-*H*-thiazolo[3,2-a]pyrimidine-6-carbonitrile (**9a**) was crystallized from acetic acid as yellow fine crystals, yield, 63%, 2.46 g, m.p. 233–235 °C. ^1^H-NMR: δ (ppm) 5.12 (s, 1H, pyrimidine H-5), 6.81 (s, 2H, NH_2_, D_2_O exchangeable), 7.23–7.89 (m, 10H, 9 Ar–H + methine–H). ^13^C-NMR: δ (ppm) 46.6 (pyrimidine C-5), 71.8 (pyrimidine C-6), 113.6 (C-2), 116.3 (CN), 126.4, 127.1, 129.2, 133.3, 136.1, 142.0, 144.5 (aromatic carbons + methine C), 157.4, 159.2 (C-8a + C-7) and 165.5 (C=O). IR (KBr) ʋ: 3345, 3260 (NH_2_), 3026, 2984 (CH), 2213 (CN), 1695 cm^−1^ (C=O). MS (70 eV): (M+2) *m/z* 394 (6.8%), (M+) *m/z* 392 (21.6%). Anal. Calcd. For C_20_H_13_N_4_OSCl (392.81): C (61.14%), H (3.33%), N (14.26%), S (8.16%), Cl (9.02%); Found: C (61.03%), H (3.12%), N (13.92%), S (7.87%), Cl (8.63%).

7-Amino-5-(4-chloropenyl)-2-(4-methoxyphenylyl)methylene-3-oxo-2,3-dihydro-5-*H*-thiazolo[3,2-a]pyrimidine-6-carbonitrile (**9b**) was crystallized from dioxane as deep yellow crystals, yield, 70%, 2.87 g, m.p. 245–246 °C. ^1^H-NMR: δ (ppm) 3.41 (s, 3H, OCH_3_), 5.07 (s, 1H, pyrimidine H-5), 7.20–7.67 (m, 9H, 8 Ar–H + methine-H), 8.3 (s, 2H, NH_2_, D_2_O exchangeable). ^13^C-NMR: δ (ppm) 48.4 (pyrimidine C-5), 61.1 (OCH_3_), 69.8 (pyrimidine C-6), 113.3 (C-2), 116.4 (CN), 126.7, 126.9, 128.2, 131.5, 136.1, 141.2, 145.3 (aromatic carbons + methine C), 157.1, 158.8 (C-8a + C-7) and 165.3 (C=O). IR (KBr) ʋ: 3350, 3240 (NH_2_), 3024, 2985 (CH), 2213 (CN), 1688 cm^−1^ (C=O). MS (70 eV): (M+2) *m/z* 424 (12.2%), (M+) *m/z* 422 (35.4%). Anal. Calcd. For C_21_H_15_N_4_O_2_SCl (422.82): C (59.64%), H (3.57%), N (13.24%), S (7.58%), Cl (8.38%); Found: C (59.12%), H (3.11%), N (12.93%), S (7.13%), Cl (7.76%).

#### Reaction of 1a with chloroacetic acid: formation of **10**

A solution of **1a** (2.64 g; 0.01 mol) in glacial acetic acid (40 mL) containing 1.0 g fused sodium acetate was heated under reflux for 3 h with 0.95 g (0.01 mol) of chloroacetic acid. The reaction mixture was then poured onto water and the precipitated solid was filtered off, dried and recrystallized from dioxane as yellow crystals, yield, 43%, 1.35 g, m.p. 257–258 °C. ^1^H-NMR: δ (ppm) 3.6 (s, 2H, CH_2_), 5.0 (s, 1H, pyrimidine H-4), 7.21–7.70 (m, 5H, 4 Ar–H +NH, D_2_O exchangeable), 8.21 (s, 2H, NH_2_, D_2_O exchangeable), 13.10(s, 1H, OH, D_2_O exchangeable). ^13^C-NMR: δ (ppm) 41.2 (CH_2_), 48.4 (pyrimidine C-4), 69.8 (pyrimidine C-5), 116.8 (CN), 126.6, 127.9, 131.8, 141.2 (Ar–C), 159.8 (C-6) and 165.7 (C=O). IR (KBr) ʋ: 3385–3220 (br, OH, NH), 2216 (CN), 1706 cm^−1^ (C=O). MS (70 eV): (M+2) *m/z* 324 (4.8%), (M+) *m/z* 322 (16.4%). Anal. Calcd. For C_13_H_11_N_4_O_2_SCl (322.71): C (48.38%), H (3.43%), N (17.35%), S (9.93%), Cl (10.98%); Found: C (48.11%), H (3.12%), N (17.03%), S (9.13%), Cl (10.17%).

#### 7-Amino-5-(4-chloropenyl)-3-oxo-2,3-dihydro-5-*H*-thiazolo[3,2-a]pyrimidine-6-carbonitrile (**11**)

*Method A* A solution of **1a** (2.64 g; 0.01 mol) in glacial acetic acid (20 mL)/acetic anhydride (8 mL) containing 1.0 g fused sodium acetate was treated with 0.95 g (0.01 mol) chloroacetic acid and refluxed in water bath for 3 h. The reaction mixture was then cooled and the precipitated solid was filtered off, dried and recrystallized from acetic acid as yellow crystals.

*Method B* A solution of 10 (3.22 g, 0.01 mol) in glacial acetic acid (20 mL)/acetic anhydride (8 mL) was refluxed in water bath for 3 h. The reaction mixture was then cooled and the precipitated solid was filtered off and dried. The obtained solid was found to be identical in all aspects (IR, m.p., mixed m.p.) with compound 11. Yield, 61%, 1.83 g, m.p. 223–225 °C. ^1^H-NMR: δ (ppm) 3.8 (s, 2H, CH_2_), 5.3 (s, 1H, pyrimidine H-4), 7.23 (d, 2H, Ar–H, *J *= 7.5 Hz), 7.41 (d, 2H, Ar–H, *J *= 7.5 Hz), 7.88 (s, 2H, NH_2_, D_2_O exchangeable). ^13^C-NMR: δ (ppm) 34.2 (CH_2_), 48.1 (C-5), 71.8 (C-6), 116.8 (CN), 126.6, 128.6, 131.8, 141.2 (Ar–C), 159.8 (C-8a), 162.3 (C-7) and 166.5 (C=O). IR (KBr) ʋ: 3345, 3230 (NH_2_), 3020, 2895 (CH), 2215 (CN), 1695 cm^−1^ (C=O). MS (70 eV): (M+2) *m/z* 306 (10.2%), (M+) *m/z* 304 (33.4%). Anal. Calcd. For C_13_H_9_N_4_OSCl (304.71): C (51.23%), H (2.97%), N (18.38%), S (10.52%), Cl (11.63%); Found: C (50.87%), H (2.77%), N (17.93%), S (10.10%), Cl (11.07%).

### Antimicrobial screening

The newly prepared compounds were verified for their antimicrobial activity against: (a) Gram-negative: *Escherichia coli* and *Pseudomonas putida*; (b) Gram-positive: *Bacillus subtilis* and *Streptococcus lactis*; (c) fungi: *Aspergillus niger* and *Penicillium* sp.; (d) yeast: *Candida albicans.*

*Media* Three media types were used:

- *Media (1)* For bacteria (Nutrient Medium), consisting of (g/L distilled water): peptone, 5 and meat extract, 3. at pH 7.0.
- *Media (2)* For fungi (Potato Dextrose Medium), consisting of (g/L distilled water): Infusion from potatoes, 4 and D (+) glucose, 20. at pH 5.5.
- *Media (3)* For yeast (Universal Medium), consisting of (g/L distilled water): yeast extract, 3; malt extract, 3; peptone, 5 and glucose, 10 at pH 5.5.

For solid media, 2% agar was added. All media were sterilized at 121 °C for 20 min. Procedure (Filter Paper Diffusion Method) [42]. Suitable concentrations of microbial suspensions were prepared from (1 for bacteria to 3 for yeast and fungi)-day-old liquid stock cultures in cubated on a rotary shaker (100 rpm). In the case of fungi, 5 sterile glass beads were added to each culture flask. The mycelia were then subdivided by mechanical stirring at speed No. 1 for 30 min. Turbidity of microorganisms was adjusted with a spectrophotometer at 350 nm to give an optical density of 1.0. Appropriate agar plates were aseptically surface inoculated uniformly by a standard volume (ca. 1 mL) of the microbial broth culture of the tested microorganism, namely *E. coli*, *P. putida*, *B. subtilis*, *S. lactis*, *A. niger*, *Penicillium* sp. and *C. albicans*. Whatman No. 3 filter paper discs of 10 mm diameter were sterilized by autoclaving for 15 min at 121 °C. Test compounds were dissolved in 80% ethyl alcohol (final concentrations are ~ 70% ethanol, ~ 5% methanol and ~ 5% isopropanol. Contains ~ 20% water) to give final concentration of 5 mg/mL. The sterile discs were impregnated with the test compounds (50 μg/disc). After the impregnated discs have been air dried, they were placed on the agar surface previously seeded with the organism to be tested. Discs were gently pressed with forceps to insure thorough contact with the media. Three discs were arranged per dish, suitably spaced apart, i.e., the discs should be separated by a distance that is equal to or slightly greater than the sum of the diameters of inhibition produced by each disc alone. Each test compound was conducted in triplicate. Plates were kept in the refrigerator at 5 °C for 1 h to permit good diffusion before transferring them to an incubator at 37 °C for 24 h for bacteria and at 30 °C for 72 h for yeast and fungi [32].

## Conclusions

New polycyclic fused pyrimidines have been synthesized using both conventional methods and microwave assisted conditions. The latter methods proved very efficient in reducing reaction times, minimization of energy consumption, management of analytical waste and increased safety for the operator as well as better reaction yields. All prepared compounds were verified for their antimicrobial activities. Some compounds showed moderate or weak antimicrobial activity. The absorption and fluorescence emission of some of the prepared compounds were studied in dioxane.
